# CD107a Expression and IFN-γ Production as Markers for Evaluation of Cytotoxic CD3+ CD8+ T Cell Response to CMV Antigen in Women with Recurrent Spontaneous Abortion

**Published:** 2013-12-22

**Authors:** Batoul Tarokhian, Roya Sherkat, Mohamma Hossein Nasr Esfahani, Minoo Adib, Abbas Kiani Esfahani, Behrooz Ataei

**Affiliations:** 1 Acquired Immunodeficiency Research Center, Isfahan University of Medical Sciences, Isfahan, Iran; 2 Department of Reproduction Biotechnology at Reproductive Biomedicine Research Center, Royan Institute for Biotechnology, ACECR, Isfahan, Iran; 3 Department of Immunology, Isfahan University of Medical Sciences, Isfahan, Iran; 4 Infectious Diseases and Tropical Medicine Research Center, Isfahan University of Medical Sciences, Isfahan, Iran

**Keywords:** Cytotoxic T Lymphocyte, CMV, Recurrent Abortion

## Abstract

**Background:**

Some evidence has shown a relationship between primary human cytomegalovirus (CMV) infection and pregnancy loss. The impact of CMV infection reactivation during pregnancy on adverse pregnancy outcomes is not completely understood. It is proposed that altered immune response, and therefore, recurrence or reactivation of latent CMV infection may relate to recurrent spontaneous abortion (RSA); however, few data are available in this regard. To find out about any cell mediated defect and reactivation of latent CMV infection in women with RPL, cellular immunity to the virus has been evaluated by specific cytotoxic T lymphocyte (CTL) response to CMV.

**Materials and Methods:**

In a case control study, CTL CD107a expression and in- tercellular IFN-γ production in response to CMV pp65 antigen and staphylococcus enterotoxin B (SEB) in women with RSA were assessed by flow cytometric analysis. Forty-four cases with history of recurrent pregnancy and forty-four controls with history of successful pregnancies were included. The FACSCaliber flow cytometer were used for analysis.

**Results:**

No significant difference was observed between CD107a expression and IFN-γ production in response to CMV PP65 antigen in RPL patients and control group. How- ever, the cytotoxic response to SEB antigen in patients with RPL was significantly lower than control group (p=0.042).

**Conclusion:**

The results of this study show that impaired CD107a expression and IFN-γ production as CTL response to CMV does not appear to be a major contrib- uting and immune incompetence factor in patients with RPL, but cytotoxic T cell response defect to other antigens requires to be assessed further in these patients.

## Introduction

Cytomegalovirus (CMV), as a member of the human herpes virus family, resides in the host throughout life without causing any symptoms in immunocompetent individuals; therefore, 50-90% of the populations have become seropositive by adulthood (1). Clinical manifestations are various, but symptomatic disease is rare among immunocompetent hosts. Severe scenarios mostly occur in immunocompromised hosts and pregnant women, so recurrent spontaneous abortion (RSA) is considered as one of the most frustrating and complex issues in reproductive medicine (2).

The etiology is still unclear and few evidencebased diagnostic and treatment approaches are available (2). Etiologic factors associated with RSA are suggested as anatomical, immunological, genetic, endocrine, infectious, thrombophilic, and environmental factors (3, 4).

Despite numerous reports between association of CMV and pregnancy loss, the role of CMV in RSA remains to be elucidated (5).

The role of altered immune response, and therefore, recurrence or reactivation of latent CMV infection may relate to RSA is unclear, whereas few data are available in this regard.

To find out the probability of any relation between recurrence or reactivation of latent CMV infection and immunological deficit and RSA, cellular immunity to CMV has been assessed by CMV-specific cytotoxic T lymphocyte (CTL) response. CMV-specific CD8 T-cells have two main effector functions: cytotoxic response and cytokine production (6). The aims of this study were to assess these main effector functions by assessment of a granule-dependent (perforin/granzyme) pathway of Cytotoxic CD3+ CD8+ T lymphocytes (CTL) using CD107a expression, lysosomal associated membrane glycoproteins (LAMPs), that are expressed on cell surface of cytotoxic T cells during degranulation and intercellular IFN-γ production.

## Materials and Methods

This case control study included forty-four cases with history of unexplained RSA before 20 weeks of pregnancy, and forty-four controls with history of successful pregnancies and no history of abortion with the mean age of 30.52 ± 4.43 and 29.45 ± 4.83 years, respectively. The studied couples were healthy with normal karyotypes. The anatomical, endocrine and metabolic disorders also known as immunodeficiency and autoimmune diseases were ruled out in cases and controls.

### Antibodies and reagent

The monoclonal antibodies and other materials used in this study include: anti-CD8 (FITC), anti-CD107a (PE), anti-IFN-γ (PE), antiCD3 (PerCp), Isotype control IgG1κ/ IgG1κ (PE/FITC), IgG1κ (PerCP) and (anti- CD28 antibodies were obtained from BDBioscience (USA) cytomegalovirus PP65 antigen . The monoclonal antibodies and other materials used in this study include: anti-CD8 (FITC), anti-CD107a (PE), anti-IFN-γ (PE), antiCD3 (PerCp), Isotype control IgG1κ/ IgG1κ (PE/FITC), IgG1κ (PerCP) and anti- CD28 antibodies were obtained from BD-Bioscience (USA), cytomegalovirus PP65 antigen (CMV PP65 Antigen; Milteny-biotec, Germany), staphylococcus enterotoxin B (SEB; Sigma, USA). Hypaque-Ficoll (Innotrain, Germany). RPMI 1640 medium with L-glutamine and sodium bicarbonate, penicillin/streptomycin and fetal calf serum (FCS) were obtained Sigma-Aldrich, USA. Fixation/Permeabilization kit with BD Golgi-Plug protein transport inhibitor, containing brefeldin A, was obtained from BD pharmingen, USA, while IgM and IgG anti-CMV kits were obtained from Radim, Italy.

### Cell isolation and culture

Fresh peripheral blood mononuclear cells (PBMCs) were isolated using Hypaque-Ficoll (Innotrain, Germany) density centrifugation. Upon wash with phosphate buffered saline (PBS), 106 PBMCs were resuspended in 1-ml volume of RPMI 1640 medium with 2 mM/L of L-glutamine and sodium bicarbonate (Sigma-Aldrich, USA) supplemented with 100 U/ml penicillin, 100 mg/ ml streptomycin and 10% heat inactivated fetal calf serum (FCS).

### Cell stimulation and staining for CD107a expression

The cells were stimulated with 1 mg/ml of anti-CD28 and 1 ml of CMV PP65 Antigen according to the manufacturer’s instructions. Conjugated Ab to CD107a (PE) was added to the cells before stimulation (7). In all experiments, staphylococcus enterotoxin B (SEB, 1 mg/ml) and anti-CD28 were used as a positive control and a negative control for spontaneous expression of CD107a, respectively. Duration of cultures was 5 hours at 37˚C in a 5% CO_2_ incubator. Then, cells were washed twice with PBS and were stained using conjugated antibodies, anti-CD3 [peridinin chlorophyll protein (PerCP)] and anti-CD8 [fluorescein isothiocyanate (FITC)], for 15 minutes at room temperature. Then the cells were washed, fixed with 1% paraformaldehyde, and analyzed on the FACSCalibur flow cytometer (Becton-Dickinson, USA).

### Cell stimulation and staining for IFN-γ production

Stimulation was carried out with 1 mg/ml of anti-CD28, and 1 ml of CMV PP65 antigen according to the manufacturer’s recommendation. In all experiments, SEB and anti-CD28 were used as a positive control and a negative control to account for spontaneous production of cytokine, respectively. The first duration of culture was 1 hour at 37˚C in a 5% CO_2_ followed by addition of 1 ml of inhibitor brefeldine A (Golgy plug; BD pharmingen, USA) for further 4 hours. Then, cells were washed twice with PBS, and were stained using conjugated antibodies, anti-CD3 (perCP) and anti-CD8 (FITC), for 15 minutes at room temperature. Following surface staining, the cells were fixed and permeabilized for 20 minutes at 4˚C using 250 ml of Cytofix/Cytoperm solution. Then, cells were washed twice with 1 ml Perm/Wash solution. Next, PE-conjugated anti-IFN-γ was used to stain PBMCs for 30 minutes at 4˚C. Corresponding isotype controls were utilized for control staining. After intracellular staining, the cells were washed twice with 1 m Perm/ Wash solution, resuspended in 0.5 ml of 1% paraformaldehyde, and analyzed on the FACSCalibur flow cytometer (Becton-Dickinson, USA).

### Flow cytometric analysis

**Fig 1:**
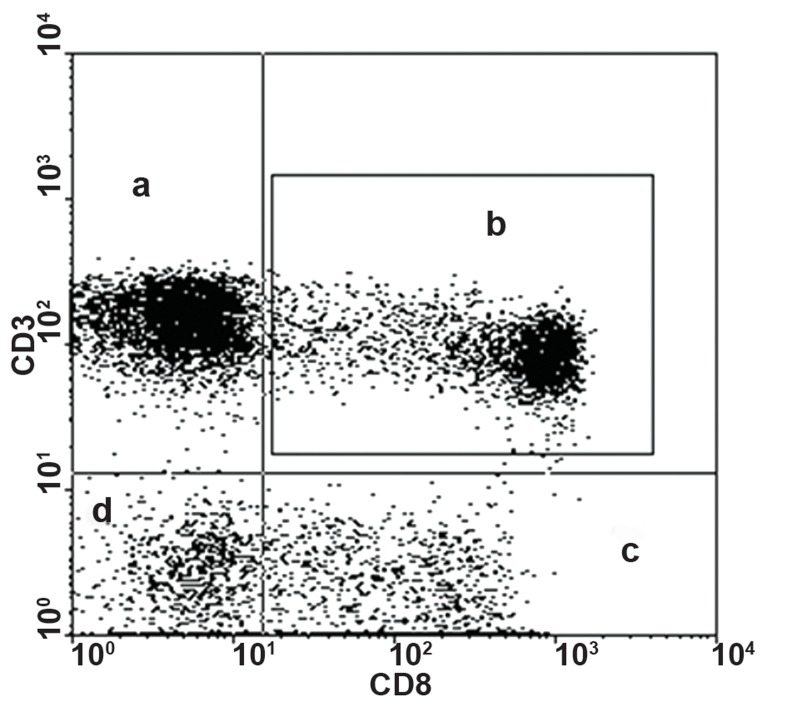
Cytotoxic T-cell population using anti-CD3 (PerCP) and anti-CD8 (FITC). Each plot has been divided into four quadrants, each of which is representative of cells separated by related flourecent dye. a. CD3 single positive (PerCP), b. CD3/CD8 double positive (PerCP/FITC), c. CD8 single positive (FITC), d. double negative.

**Fig 2:**
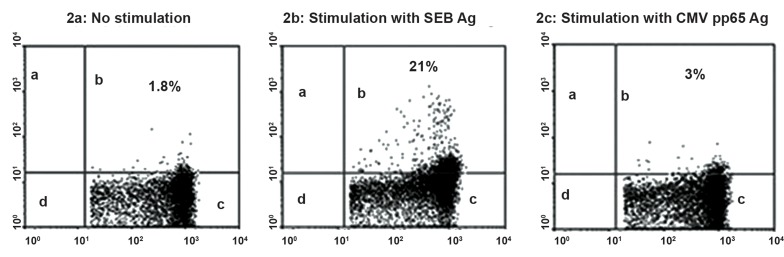
Typical flow cytometry for CD107a expression on CD3+ CD8+ T cells.

**Fig 3:**
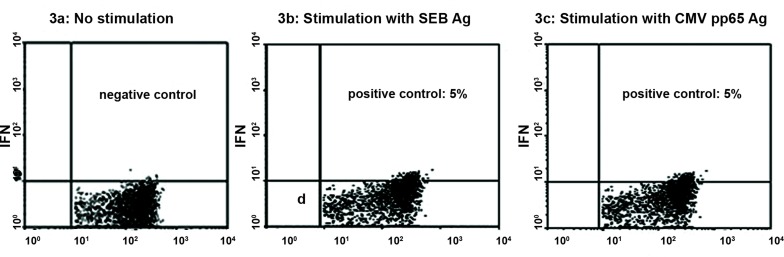
IFN-γ production in CD3+ CD8+ T cells.

Three-color flow cytometry analysis was performed on a FACSCalibur flow cytometer using Windows Multiple Document Interface (WinMDI) software. The gate was set around the lymphocytes to exclude other cells from analysis. The isotype IgG control was used for background control. Fluorescence from FL1 [fluorescein isothiocyanate (FITC)], FL2 [phycoerythrin (PE)] and FL3 [peridinin chlorophyll protein (PerCP)] channels were utilized to measure cell surface antigens and intracellular cytokine. Forward versus side scatter was obtained to analyze the lymphocyte population. Anti-CD3 (PerCP) and anti-CD8 (FITC) were used to identify cytotoxic T-cell population (Fig 1). All data were expressed as the percentage of CD107a positive on CD3+ and CD8+ cells (Fig 2 a, b, c), and the percentage of IFN-γ positive on CD3+ and CD8+ cells (Fig 3 a, b, c).

### Human cytomegalovirus serology

The enzyme-linked immunosorbent assay (ELISA) technique was performed using kits (Radim, Italy) intended for estimating concentration of specific anti-CMV IgG and for detecting specific anti-CMV IgM. The techniques were performed according to the manufacturer’s instructions.

### Statistical analyses

An independent-samples T test was used to compare CD107a expression and IFN-γ production in CD8+ CD3+ T cells in women with RSA and control group.

Pearson correlation coefficient was used to determine correlation between CD107a expression, IFN-γ production and anti-CMV IgG in women with RSA and control group. The value of p<0.05 was considered significant.

### Ethical aspects

The subjects in this study were enrolled voluntarily after being given a brief description of the purpose and procedure of the study and after signing a written informed consent form. This project was approved by the Ethical Committee of Isfahan University of Medical sciences.

## Results

Specific cytotoxic response to CMV was evaluated by CD107a expression on CD3+ CD8+ T cell in response to CMV PP65 antigen in RSA patients and controls. No significant difference was observed between the expression of CD107a on CD3+CD8+ T cells in cases and controls (2.63 ± 1.18 vs. 2.78 ± 1.43; p=0.29).

However, the cytotoxic response to SEB antigen in RSA patients was significantly lower than control group (11.17 ± 6.09 vs. 13.73 ± 4.95; p=0.042, Table 1), but CD3+CD8+ T cell IFN-γ production in response to CMV PP65 (0.64 ± 0.91 vs. 0.62 ± 0.70; p=0.89) and SEB antigen (4.71 ± 0.43 vs. 4.44 ± 1.07; p=0.22) in RSA patients and control group was not significantly different (Table 2).

Percentages of CD8 positive cells in RSA women were higher than control group (6.52 ± 4.12 vs. 5.39 ± 2.81 p=0.08). This difference was close to be statistically significant (Table 3).

CMV specific IgM antibody was negative in all of RSA patients and controls. About 97.73% of individuals in both groups were seropostive for CMV IgG (Table 4).

In addition, results showed no significant difference between concentration of anti-CMV IgG in RSA and control group (182.91 ± 74.89 vs. 190.19 ± 70.54; p=0. 32).

No significant correlation among anti-CMV IgG, CD107a, and IFN-γ in RSA and control group were found (Table 5).

**Table 1:** Comparison of CD107 expression in response to CMV PP65 and SEB antigen on CD3 + CD8+ T cells between control group and RSA patients


Variant	RSA		Control		p-value
	Mean(SD)	(Min, Max)	Mean(SD)	(Min, Max)		

**CD107a expression (Fig response to CMV PP65)**	2.63 ± 1.18	0.6, 5.5	2.78 ± 1.43	0.8, 7.2	0.29
**CD107a expression (response to SEB)**	11.17 ± 6.09	0.1, 23	13.73 ± 4.95	5.30, 22	0.042*


Results are expressed as percentage of CD107a positive cells. *; The value of p<0.05 was considered significant.

**Table 2:** Comparison of IFN-γ production in response to CMV PP65 and SEB antigen in CD3+ CD8+ T cells between control group and RSA patients


Variant	Case		Control		P value
	Mean (SD)	(Min-Max)	Mean (SD)	(Min-Max)		

**IFN-γ production response to CMV PP65 **	0.64 ± 0.91	0.1-6.1	0.62 ± 0.70	0.1-3.5	0.89
**IFN-γ production response to SEB**	4.71 ± 0.43	3.4- 5.6	4.44 ± 1.07	0.1-5.8	0.22


Results are expressed as percentage of IFN-γ positive cells.

**Table 3:** Comparison of CD8 expression on surface of lymphocytes between RSA patients and control group


Variant	RSA		Control		P value
	Mean(SD)	(Min-Max)	Mean(SD)	(Min- Max)		

**CD8**	6.52 ± 4.12	1.78-20.22	5.39 ± 2.81	0.62-12.24	0.08


Results are expressed as percentage of CD8 positive cells.

**Table 4:** Comparison of anti-CMV IgG concentration between RSA patients and control group


Variant	RSA		control		P value
	Mean ± SD	(Min-Max)	Mean ± SD	(Min-Max)		

**Anti-CMV IgG concentration (RU/ml)**	182.91 ± 74.89	73.03-291.67	190.19±70.54	75.03-282.02	0.32


**Table 5:** Correlation between anti-CMV IgG, CD107a, and IFN-γ in RSA and control group


Variant	CD107a	Anti–CMV IgG	IFN-γ

**CD107a**		r= -0.027P= 0.804	r=-0.065P=0.563
**Anti–CMV IgG**	r= -0.027P= 0.804		r =0.108P=0.333
**IFN-γ**	r=-0.065P=0.563	r =0.108P=0.333	


## Discussion

Few studies are available on the association between CMV infection and RSA, while controversial results have been reported (8- 10). The epithelium of the upper alimentary, respiratory, or genitourinary tracts is the first entrance site of the virus and through direct damage, induces vascular thrombosis by inhibiting anticoagulant properties and enhances coagulant properties in immunocompromised patients (11). Thrombosis can also be produced indirectly by antiphospholipid antibodies (APL) induced by CMV (5). These two mechanisms exaggerate each other in thrombosis, and the condition is induced when CMV is reactivated in immune comprised individuals. Thrombosis and aPL antidobies are identified as risk factors for recurrent abortion.

Immunological non-responsiveness has been considered as one of the underlying role of RSA, especially reported for CMV infection (8).

Humoral and cellular immunity to cytomegalovirus (CMV) has been evaluated in women with unexplained RSA. While some authors reported higher prevalence and higher antibody titers to CMV in RSA cases (6), other studies showed comparable and even a significantly lower prevalence of serum anti-CMV antibodies in RSA women compared with matched controls (9).

Additionally, lymphocyte proliferation to CMV has been prominently impaired in RSA women (9). A degree of immunological nonresponsiveness both to cytomegalovirus and to cell-surface alloantigens in habitually aborting women has been reported (10). These results could be indicative of difficulty in responding to CMV and reactivation of latent infection in women with unexplained RSA.

Despite these studies, there is no report on the cytotoxic response to CMV in RSA patients. Therefore, the present study evaluated cytotoxic response to CMV by surface expression of CD107a on CD3+ CD8+ T cell, production of IFN-γ by CD3+ CD8+ T cells and humoral response to CMV in RSA women and controls. The result revealed no significant difference between specific cytotoxic response to CMV in RSA patients and control group, indicating that the activation of latent or recurrent CMV infection due to their impaired immune function is not a risk factor for women with recurrent abortion.

Shimada et al. (12) found the percentages of CD3+ cells, CD4+ IFN-γ+ cells and CD4+ TNF-α+ cells were significantly lower in the endometrium of RSA women compared with control women. They further showed that the percentages of lymphocytes and CD3+ cells were significantly lower in RSA women than controls, while the percentages of CD4+ or CD8+ cells were similar; in addition, percentage of CD4+ IFN-γ+ and CD8+ IFN-γ+ cells in RSA women were insignificantly lower than control (13). In our study, IFN-γ production by CD3+ CD8+ T cells in response to CMV antigen was not significantly different between RSA patient and healthy controls. These results also ruled out the impaired immune function to CMV in RSA women which could cause the activation of latent CMV infection or its recurrent infection in these patients.

However, in our study, CD107a surface expression on CD3+ CD8+ T cells in response to SEB, as a super-antigen that could act as nonspecific stimulant for cells, was lower in RSA patient as compared to control group. This data provides a supportive issue to lower cytotoxic response evaluated by CD107a surface expression in RSA patient compared with control group, but this response is nonspecific and needs further assessment.

After assessing the number of CD8 T cells population between the RSA and control group, the finding revealed that the CD8 percentages in RSA women were insignificantly higher than those in control group, which is the same as previous studies by Michimata et al. (7) and Darmochwal- Kolarza et al. (13), whereas in contrast to reports by Malinowski et al. (14) and Lachapelle et al. (15); they found lower CD8 T lymphocyte proportion in blood and endometrium in patients with RSA, indicating that the number of CD8 T cells is not the major contributing factor for RSA.

In our study, the percentage of positive IgG titer between RSA and control group was the same (97.73%). Although the percentage of positive IgG titer was higher than reported values in literature, it is still close to the previous studies (80 to 94%), and no difference was also found between RSA and control group like previous studies. In this study, none of the individuals were IgM positive, indicating that none of the cases had primary CMV infection, so this result seems to be the same as other studies (9, 16-18). The only contradictory report in the literature belongs to Radcliffe et al. (10) in which they found lower prevalence of serum anti CMV antibodies in RSA patients compared to fertile controls.

## Conclusion

The results of this study reveal that impaired CD107a expression and IFN-γ production as cytotoxic response to CMV fails to be a major contributing factor for RSA.

Cytotoxic responses to different antigens or different component of CMV virus may have to be used in future studies. In addition, our findings reveal that the only difference between the RSA and control group is T cell response to super-antigen, which requires further evaluation in RSA patients.
